# Cannabis Use and Adverse Childhood Experiences Among Cancer Survivors

**DOI:** 10.1002/cam4.71400

**Published:** 2025-11-23

**Authors:** May Z. Gao, Oluwole A. Babatunde, Melanie S. Jefferson, Swann A. Adams, Chanita Hughes Halbert, Nosayaba Osazuwa‐Peters, Eric Adjei Boakye

**Affiliations:** ^1^ Duke University School of Medicine Durham North Carolina USA; ^2^ Prisma Health Psychiatry Greer South Carolina USA; ^3^ Department of Psychiatry Medical University of South Carolina Charleston South Carolina USA; ^4^ College of Nursing and the Department of Epidemiology & Biostatistics University of South Carolina Columbia South Carolina USA; ^5^ University of Southern California Los Angeles California USA; ^6^ Department of Head and Neck Surgery & Communication Sciences Duke University School of Medicine Durham North Carolina USA; ^7^ Department of Population Health Sciences Duke University School of Medicine Durham North Carolina USA; ^8^ Duke Cancer Institute Durham North Carolina USA; ^9^ Department of Public Health Sciences Henry Ford Health System Detroit Michigan USA; ^10^ Department of Otolaryngology—Head and Neck Surgery, Henry Ford Health System Detroit Michigan USA; ^11^ Henry Ford Health + Michigan State University Health Sciences Detroit Michigan USA; ^12^ Department of Epidemiology and Biostatistics Michigan State University College of Human Medicine East Lansing Michigan USA

**Keywords:** adverse childhood experiences, cancer, cancer survivorship, cannabis, substance use disorder

## Abstract

**Objective:**

To examine the association between adverse childhood events (ACEs) and cannabis use among adult cancer survivors in the United States.

**Methods:**

We conducted a cross‐sectional study of cancer survivors ≥ 18 years old using 2020 Behavioral Risk Factor Surveillance System data. ACEs were categorized as 0, 1, 2–3, and ≥ 4. Weighted multivariable logistic regression estimated the odds of cannabis use by ACE category.

**Results:**

Among 7896 cancer survivors, cannabis use prevalence was 6.0%. ACE distribution was 44.1% (0), 22.7% (1), 20.2% (2–3), and 13.0% (≥ 4). Cannabis use was more common among younger adults, Hispanics, never‐married individuals, smokers, and those reporting fair/poor health. Compared to those with 0 ACEs, cancer survivors with 2–3 ACEs (aOR: 2.56, 95% CI: 1.57–4.27) and ≥ 4 ACEs (aOR: 4.10, 95% CI: 2.54–6.64) had significantly higher odds of cannabis use.

**Conclusions:**

Cancer survivors with a higher number of ACEs reported increased odds of cannabis use. These findings support further study of ACEs and substance use in cancer survivors and may inform trauma‐informed survivorship care.

## 1 Introduction

Adverse childhood experiences (ACEs) encompass a broad range of potentially traumatic events occurring before the age of 18, including abuse, neglect, and household dysfunction [1]. Across studies conducted among cancer patients in the United States, the prevalence of ACEs ranges anywhere from 40% to 59% [2]. Prior literature has consistently demonstrated that ACEs are strongly linked to negative health outcomes in adulthood, including mental health disorders, chronic diseases, and engagement in high‐risk behaviors such as substance use [3, 4, 5]. A meta‐analysis showed that individuals with ≥ 4 ACEs had a higher likelihood of adverse health behaviors related to substance use, mental health, and physical health outcomes [6]. The potentially significant burden and impact of ACEs on lifelong health underscores their significance as a public health concern.

While associations between ACEs and substance use have been extensively studied in the general population [1, 7], the association between ACEs and cannabis use among cancer survivors is unclear. Cancer survivorship presents unique challenges, including physical pain, psychological distress, and long‐term health complications associated with treatment [8]. This raises the concern that cancer survivors with high ACE exposure may be more vulnerable to cannabis use, whether for therapeutic or recreational purposes. As cannabis use becomes increasingly prevalent due to policy shifts toward legalization and decriminalization in many states [9, 10], its role in cancer care is gaining attention. Cannabis is utilized by cancer patients for symptom management, particularly for pain relief, nausea, and appetite stimulation [11]. However, concerns remain regarding potential risks, including cognitive impairment, respiratory issues, and the lack of standardized clinical guidelines for its use in oncology [11].

Understanding cannabis use in cancer survivors, particularly among those with ACEs, is important given that ACEs have been associated with poorer health outcomes and increased stress‐related disorders [3, 6]. This study examined the association between ACEs and cannabis use among adult cancer survivors in the United States.

## 2 Methods

### 2.1 Data Source

We used data from the 2020 Behavioral Risk Factor Surveillance System (BRFSS). The BRFSS is an annual cross‐sectional survey that provides nationally and state‐representative self‐reported data on demographic characteristics, health, and health behaviors for noninstitutionalized U.S. civilians aged 18 years and older. The BRFSS uses a random‐digit–dialed telephone survey overseen by the Centers for Disease Control and Prevention (CDC) and reaches over 500,000 respondents. The BRFSS has 3 overall components: core modules (sets of questions consistently administered to all states and territories to establish national estimates), optional modules (CDC–developed questions that states can include in their BRFSS survey depending on their priorities), and state‐added questions (state‐customized items). Consistent with the Common Rule, this cross‐sectional study of deidentified publicly available BRFSS data did not require institutional review board approval or informed consent.

In 2020, 28 states (AL, AZ, CA, DC, FL, GA, HI, ID, IA, KS, KY, MD, MA, MS, MO, MT, NV, NJ, ND, OK, RI, SC, SD, TX, UT, VA, WI, WY) included state‐added ACE questions. In this study, we included adult cancer survivors who were identified with a yes answer to the question “Ever been told you have skin cancer or melanoma or any other types of cancer?”

### 2.2 Measures

The exposure was ACE which is made up of three forms of abuse (physical, emotional, and sexual) and five types of household challenges, such as having family members with substance misuse, incarceration, or mental illness; parental divorce; and witnessing intimate partner violence. A *yes* response to each question was coded as one, and the scores were summed to generate a total ACE score, ranging from 0 to 11 [12]. We categorized ACEs into four levels: 0, 1, 3–3, and ≥ 4. The outcome was self‐reported cannabis use (yes or no). Covariates in the study included self‐reported age at survey, gender, race/ethnicity, marital status, educational level, income level, BMI, cigarette smoking status, general health status and depression.

### 2.3 Statistical Analysis

Descriptive statistics (frequencies and weighted percents) were generated to examine the characteristics of the study sample. In addition, differences between characteristics by cannabis use were compared using chi‐square tests. A weighted multivariable logistic regression model using listwise deletion examined the association between the number of ACEs and cannabis use, adjusting for sociodemographic and health factors. Statistical tests were two‐tailed, and *p* < 0.05 was considered statistically significant using SAS v9.4 software to account for the complex sampling design of the BRFSS. Data analysis was performed between April and June 2024.

## 3 Results

A total of 7896 cancer survivors were included in the study. Cannabis use prevalence in our sample was 6.0%, with 44.1%, 22.7%, 20.2%, and 13.0% reporting 0, 1, 2–3, and ≥ 4 ACEs, respectively (Table 1). Overall, there was equal distribution across gender and BMI categories; most respondents were non‐Hispanic White (86.6%) and married (63.7%). In the unadjusted chi‐square tests, cannabis use was more prevalent among younger cancer survivors aged 18–39, Hispanic and Non‐Hispanic other survivors, those never married, current smokers, and those reporting fair/poor health. In the unadjusted bivariate analyses, we found significant differences in cannabis use across ACE categories (*p* < 0.0001), with higher ACE scores associated with increased cannabis use. In the adjusted model, cancer survivors with 2–3 ACEs (aOR: 2.56, 95% CI: 1.57–4.27), or ≥ 4 ACEs (aOR: 4.10, 95% CI: 2.54–6.64) had higher odds of cannabis use compared to those with 0 ACEs (Table 2).

**TABLE 1:** Characteristics of study sample, overall and stratified by cannabis use, 2020 Behavioral Risk Factor Surveillance System.

	Frequency (weighted %)			*p*
	Overall (*n* = 7896)	Cannabis use		
		Yes (*n* = 473)	No (*n* = 7423)	
Age at survey				< 0.0001
18–39	258 (6.3)	47 (21.4)	211 (78.6)	
40–64	2229 (36.7)	212 (8.3)	2017 (91.7)	
≥ 65	5305 (57.0)	210 (3.0)	5095 (97.0)	
Gender				0.1242
Female	4452 (54.5)	258 (6.2)	4194 (93.8)	
Male	3444 (45.5)	215 (5.9)	3229 (94.1)	
Race/ethnicity				< 0.0001
Non‐Hispanic White	6874 (86.8)	378 (5.2)	6496 (94.8)	
Non‐Hispanic Black	278 (5.3)	17 (9.5)	261 (90.5)	
Hispanic	150 (1.9)	23 (19.4)	127 (80.6)	
Non‐Hispanic other	594 (5.9)	55 (11.3)	539 (88.7)	
Marital status				0.0028
Married	4680 (63.7)	237 (5.5)	4443 (94.5)	
Divorced/separated/widowed	2689 (29.9)	171 (5.8)	2518 (94.2)	
Never married	491 (6.4)	62 (11.9)	429 (88.1)	
Education level				0.2588
College graduate	3225 (27.7)	179 (5.5)	3046 (94.5)	
Some college	2417 (33.9)	166 (7.2)	2251 (92.8)	
≤ High school graduate	2241 (38.4)	128 (5.6)	2113 (94.4)	
Income level				0.0726
≥ 50 K	3383 (40.3)	185 (5.6)	3198 (94.4)	
25–50 K	1706 (20.6)	109 (6.0)	1597 (94.0)	
< 25 K	1328 (20.4)	120 (8.4)	1208 (91.6)	
Refused or missing	1479 (18.7)	59 (4.7)	1420 (95.3)	
BMI category				0.1540
Normal	2513 (30.0)	180 (6.0)	2333 (94.0)	
Overweight	2770 (36.3)	162 (6.0)	2608 (94.0)	
Obese	2192 (33.7)	115 (6.4)	2077 (93.6)	
Smoking status				< 0.0001
Never	4445 (50.8)	157 (3.3)	4288 (96.7)	
Former	2662 (34.2)	205 (7.5)	2457 (92.5)	
Current	741 (15.0)	111 (12.5)	630 (87.5)	
General health status				0.0150
Excellent/very good	3443 (39.9)	192 (5.0)	3251 (95.0)	
Good	2560 (32.1)	124 (5.5)	2436 (94.5)	
Fair/poor	1893 (28.0)	157 (8.3)	1736 (91.7)	
Self‐reported depression				< 0.0001
Yes	1509 (22.6)	159 (10.2)	1350 (89.8)	
No	6364 (77.4)	313 (4.9)	6051 (95.1)	
Number of ACEs				< 0.0001
0	3481 (39.9)	99 (2.6)	3382 (97.4)	
1	1790 (22.1)	94 (4.3)	1696 (95.7)	
2–3	1599 (21.5)	125 (7.2)	1474 (92.8)	
> 4	1026 (16.5)	155 (15.4)	871 (85.6)	

Abbreviation: ACE = Adverse childhood experiences.

**TABLE 2:** Multivariable logistic regression model^a^ estimating association between cannabis use and ACEs, 2020 Behavioral Risk Factor Surveillance System.

	Odd ratio (95% CI)
Number of ACEs	
0	Reference
1	1.55 (0.92, 2.60)
2–3	2.56 (1.57, 4.27)
> 4	4.10 (2.54, 6.64)
Age at survey	
18–39	4.99 (2.46, 10.11)
40–64	2.17 (1.48, 3.19)
≥ 65	Reference
Gender	
Female	0.87 (0.61, 1.23)
Male	Reference
Race/ethnicity	
Non‐Hispanic White	Reference
Non‐Hispanic Black	1.79 (0.86, 3.74)
Hispanic	2.49 (1.13, 5.48)
Non‐Hispanic other	1.76 (1.03, 2.99)
Marital status	
Married	Reference
Divorced/separated/widowed	1.05 (0.72, 1.53)
Never married	1.21 (0.67, 2.17)
Education level	
College graduate	Reference
Some college	1.02 (0.67, 2.17)
≤ High school graduate	0.67 (0.40, 1.10)
Income level	
≥ 50 K	Reference
25–50 K	1.16 (0.71, 1.88)
< 25 K	1.17 (0.68, 2.02)
Refused or missing	1.03 (0.55, 1.92)
BMI category	
Normal	Reference
Overweight	1.10 (0.73, 1.66)
Obese	1.00 (0.62, 1.60)
General health status	
Excellent/very good	Reference
Good	1.03 (0.63, 1.67)
Fair/poor	1.51 (0.94, 2.41)
Self‐reported depression	
Yes	0.91 (0.61, 1.35)
No	Reference

Abbreviation: ACE = Adverse childhood experiences.

^a^ Model was adjusted for age, gender, race/ethnicity, marital status, education level, income level, BMI, general health status and depression.

## 4 Discussion

Cancer survivors with more ACEs had higher odds of cannabis use, consistent with broader literature associating childhood adversity with adult substance use [6, 11]. Our findings contribute to previous research [1, 3, 6, 7] by extending this association to cancer survivors, who often experience additional psychological and physical challenges that may further contribute to cannabis use [8, 11]. Prior research has consistently shown that individuals reporting multiple ACEs have a significantly higher likelihood of engaging in substance use, including cannabis, to manage stress, anxiety, and unresolved psychological distress [6, 7]. The observed association between ACEs and cannabis use underscores the need for targeted interventions for cancer survivors with a high ACE burden, as well as the importance of evidence‐based guidelines for cannabis use in oncology. The prevalence of cannabis use among cancer survivors in our cohort was 6%, compared to estimates from the literature ranging from 8% to 31% [13, 14, 15]. The relatively low prevalence of cannabis use observed in our study may reflect limited legalization across participating states in 2020 and the lag between policy change and behavior [10, 16, 17].

We found that cancer survivors with a history of ≥ 4 ACEs had higher odds of reporting cannabis use. Research suggests that early‐life stress may shape neurobiological pathways involved in reward and stress regulation, which could influence substance use behaviors later in life [3, 18]. While cannabis use may provide symptomatic relief for cancer‐related issues [11], the elevated prevalence among those with high ACEs raises concerns about potential dependence. Cancer survivors with a history of ACEs may be at an increased risk of using cannabis not solely for medical purposes but also as a means of coping with underlying psychological distress. This raises important considerations for healthcare providers, as unregulated or excessive cannabis use may contribute to dependency, exacerbate mental health conditions, or interfere with cancer treatments [15]. Despite growing interest in cannabis as a therapeutic agent, clinical guidelines in oncology remain limited due to insufficient evidence on dosing, administration, and long‐term safety [11]. The American Society of Clinical Oncology recently published guidelines cautioning that while cannabis can be beneficial for certain symptoms, its use should be carefully considered within an evidence‐based framework [8]. Therefore, it is imperative that medical professionals discuss the risks and benefits of cannabis use with patients, particularly among those with a history of ACEs. Understanding the association between childhood adversity and cannabis use may aid oncology providers in identifying patients who would benefit from early behavioral health intervention and closer monitoring of cannabis use.

Our study's observed association between ACEs and cannabis use highlights the potential benefit of screening for childhood adversity in cancer care. Identifying cancer survivors with a history of ACEs may help clinicians better recognize those at heightened risk for substance use and psychological distress, enabling earlier referral to trauma‐informed care, behavioral health services, or supportive interventions tailored to individual coping needs [6, 19]. Integrating psychological support into cancer survivorship care may also reduce reliance on cannabis for emotional regulation, especially when used outside of medical guidance. Trauma‐informed care has been suggested as a multilevel framework for addressing ACEs, including education, screening and referral, and trauma‐focused therapy [20]. However, outcome evaluation studies have been limited, especially with regard to substance use behaviors.

Future prospective studies should explore the feasibility and effectiveness of ACE screening protocols in oncology settings, as well as how trauma‐informed interventions might influence long‐term health outcomes. Additionally, studies examining the intersection of ACEs, cannabis use, and specific cancer‐related symptoms (e.g., pain, insomnia, anxiety) could help distinguish medical versus non‐medical motivations for use. Relatedly, future studies should explore whether ACE‐informed interventions can mitigate cannabis use driven by psychological distress rather than medical necessity. Finally, qualitative research may also offer insights into survivor perspectives on cannabis use and how early‐life trauma shapes coping preferences in the context of cancer survivorship.

## 5 Limitations

Our study is limited by the use of self‐reported measures, which could introduce recall and social desirability biases. While 2020 marked a turning point in cannabis legalization, not all participating states had legalized recreational or medical cannabis, which may have affected both access and willingness to disclose use [10, 16, 17]. The BRFSS relies on self‐report, which may lead to underreporting due to legal and social stigma. These factors should be considered when interpreting prevalence estimates. Additionally, the lack of detailed data on cannabis use, such as frequency, dosage, and purpose (medicinal or recreational), limits understanding of its context and implications among cancer survivors. The cross‐sectional nature of the study prevents any causal inferences between ACEs and cannabis use. It is unclear whether cannabis use is a direct consequence of ACE exposure or whether other factors mediate this relationship. While the study adjusts for sociodemographic and health‐related factors, unmeasured confounders such as trauma‐related mental health disorders, other substance use behaviors, and access to healthcare could influence the observed associations. Without longitudinal data, it is difficult to determine how cannabis use patterns change over time among cancer survivors with ACE exposure or whether this usage impacts long‐term health outcomes.

## Author Contributions

**May Z. Gao:** data curation, methodology, validation, writing – original draft, writing – review and editing. **Oluwole A. Babatunde:** conceptualization, data curation, formal analysis, methodology, validation, writing – original draft, writing – review and editing. **Melanie S. Jefferson:** data curation, methodology, validation, writing – review and editing. **Swann A. Adams:** data curation, methodology, validation, writing – review and editing. **Chanita Hughes Halbert:** data curation, methodology, validation, writing – review and editing. **Nosayaba Osazuwa‐Peters:** data curation, methodology, validation, writing – review and editing, supervision. **Eric Adjei Boakye:** conceptualization, data curation, formal analysis, methodology, validation, writing – original draft, writing – review and editing, supervision.

## Ethics Statement

Consistent with the Common Rule, this cross‐sectional study of deidentified publicly available BRFSS data did not require institutional review board approval or informed consent.

## Conflicts of Interest

N.O.‐P. has received personal fees as a scientific advisor to *Merck*. The remaining authors declare no conflicts of interest.

## Data Availability

The data is publicly available and could be assessed at the NHIS website: https://www.cdc.gov/brfss/data_documentation/index.htm. Additional requests for analytical datasets used in this study may be sent to the corresponding author.
